# Preparatory Knee Flexion-Extension Movements Enhance Rapid Sidestepping Performance in Collegiate Basketball Players

**DOI:** 10.3389/fspor.2021.670649

**Published:** 2021-05-17

**Authors:** Masahiro Fujimoto, Eri Uchida, Akinori Nagano, Mark W. Rogers, Tadao Isaka

**Affiliations:** ^1^Human Augmentation Research Center, National Institute of Advanced Industrial Science and Technology, Kashiwa, Japan; ^2^College of Sport and Health Science, Ritsumeikan University, Kusatsu, Japan; ^3^Department of Physical Therapy and Rehabilitation Science, University of Maryland School of Medicine, Baltimore, MD, United States

**Keywords:** preparatory movement, lower-limb loading, ground reaction force, biomechanics, basketball

## Abstract

Lower-limb weight-bearing load distribution in stationary standing influences the timing of rapid first step initiation of importance for functional movement activities and agility performance in sports. This study investigated the effect of pre-step lower-limb loading and unloading with preparatory knee flexion-extension movements on sidestepping performance in fifteen male collegiate basketball players. Participants performed two-choice (step limb) reaction time sidestepping under two conditions: without preparatory movements before the go cue (no-prep–NP) and with continuous alternating knee extension and flexion movements (prep–P). The reaction signal was provided at the beginning of knee extension and flexion and during these movements which corresponded with the largest and smallest loading instants and the transition states between those instants. Sidestepping performance was assessed with three-dimensional kinematic data and ground reaction forces. Step initiation onset time was significantly faster by 13–15% than the NP condition when initiated in the knee flexion phase (*p* ≤ 0.028, *r* ≥ 0.70), whereas step-limb unloading interval from step initiation to step lift-off was significantly faster by 12–15% in the knee extension phase (*p* ≤ 0.01, *r* ≥ 0.74). The preparatory movements significantly shortened step lift-off by 10–12% (*p* ≤ 0.013, *r* ≥ 0.73) and step duration by 17–21% (*p* < 0.001, *r* ≥ 0.85) with 19–22% faster step velocity (*p* < 0.001, *r* ≥ 0.84), which resulted in 14–15% shorter overall time to step landing (*p* < 0.001, *r* ≥ 0.84), irrespective of the loading phases. These results indicated that lower-limb loading with pre-step knee flexion facilitated faster step initiation, while lower-limb unloading with knee extension facilitated faster step-limb unloading, both resulting in faster step lift-off. Bilateral knee flexion-extension movements as a preparatory action could be utilized by invasion sports players to facilitate reactive stepping performance for more effective movement initiation.

## Introduction

The ability to perform alterations in body weight support between the lower limbs is a fundamental component of whole-body human movement (Patla et al., 1993; Patchay and Gahery, 2003; Shinya et al., 2009; Mille et al., 2014; Sparto et al., 2014). For example, postural transitions from bipedal to single limb stance involve lower-limb loading and unloading during lateral weight transfer that accompany a variety of goal-directed actions, including the initiation of stepping, ongoing walking, athletic agility maneuvers, and hitting and throwing sports activities (e.g., basketball, tennis, baseball, and field events) (Uzu et al., 2009; Fujii et al., 2013; Mille et al., 2014; Müller et al., 2014; Ibrahim et al., 2019). In particular, first step quickness enhances the effectiveness of offensive and defensive actions during athletics, especially in invasion sports such as basketball (Fujii et al., 2014, 2015). Accordingly, identifying approaches to augment reactive stepping performance is an important goal of sports performance training.

To rapidly lift and advance the stepping limb from a stationary standing position, body weight support must be stably redistributed between the lower limbs to allow limb withdrawal (Patla et al., 1993; Patchay and Gahery, 2003; Shinya et al., 2009; Sparto et al., 2014). Hence, interlimb neuromotor coordination of postural (single limb extension support) and intended movement (flexion-abduction limb withdrawal) actions reflected in the kinetic patterns of limb loading forces is a fundamental requirement of rapid sidestepping performance (Patla et al., 1993; Sparto et al., 2014).

Because step initiation and execution require unloading of the stepping limb and increased loading of the single stance limb, the initial conditions of limb loading preceding a step can either facilitate or impede stepping. Hence, pre-step conditions of limb loading that reduce or increase step-limb loading, respectively shorten and delay movement initiation (Patchay and Gahery, 2003; Aruin, 2006; Azuma et al., 2007; Shinya et al., 2009; Mille et al., 2014) and can affect the spatiotemporal characteristics of first step execution (Mille et al., 2014). For instance, changing the level of pre-step limb loading through continuous alteration of bilateral knee flexion-extension movements can shorten rapid step initiation onset timing during limb unloading below 80% of body weight and delay sidestepping initiation time above 120% of body weight (Fujii et al., 2013). However, in addition to those discrete loaded or unloaded conditions, flexion-extension preparatory movements also involve continuously alternating increases and decreases in limb loading between these threshold levels. Thus, time-varying pre-step phasic loading conditions could affect stepping performance, but the effect is unclear.

To further address these issues, the objective of this study was to investigate the effect of preparatory pre-step lower-limb loading and unloading during bilateral knee flexion-extension movements on sidestepping performance. We hypothesized that the pre-step lower-limb loading and unloading phases would respectively reduce and improve sidestepping performance. The performance was quantified by step-initiation characteristics—step initiation time, step-limb unloading time, and step lift-off time—and step-execution characteristics—step duration, step velocity, step length, and overall time to step landing from the visual cue.

## Materials and Methods

### Participants

A total of 15 healthy, male collegiate basketball players [age: 20.0 ± 1.1 years; height: 1.74 ± 0.04 m, body mass: 68.0 ± 6.3 kg; 9.5 ± 3.1 years of experience (mean ± *SD*)] participated in this study. Since sidestepping maneuvers are often utilized in basketball competition in an effort to evade a defender or impede an attacker (Conrad, 2014), well-practiced athletes who were accustomed to such actions were selected to minimize between-participant variability. This study was conducted in accordance with the World Medical Association's Declaration of Helsinki, and the study protocol was approved by the Ethics Committee of Ritsumeikan University Biwako-Kusatsu Campus, Japan. All participants provided written informed consent before they participated in the study.

### Instrumentation

**Figure 1:**
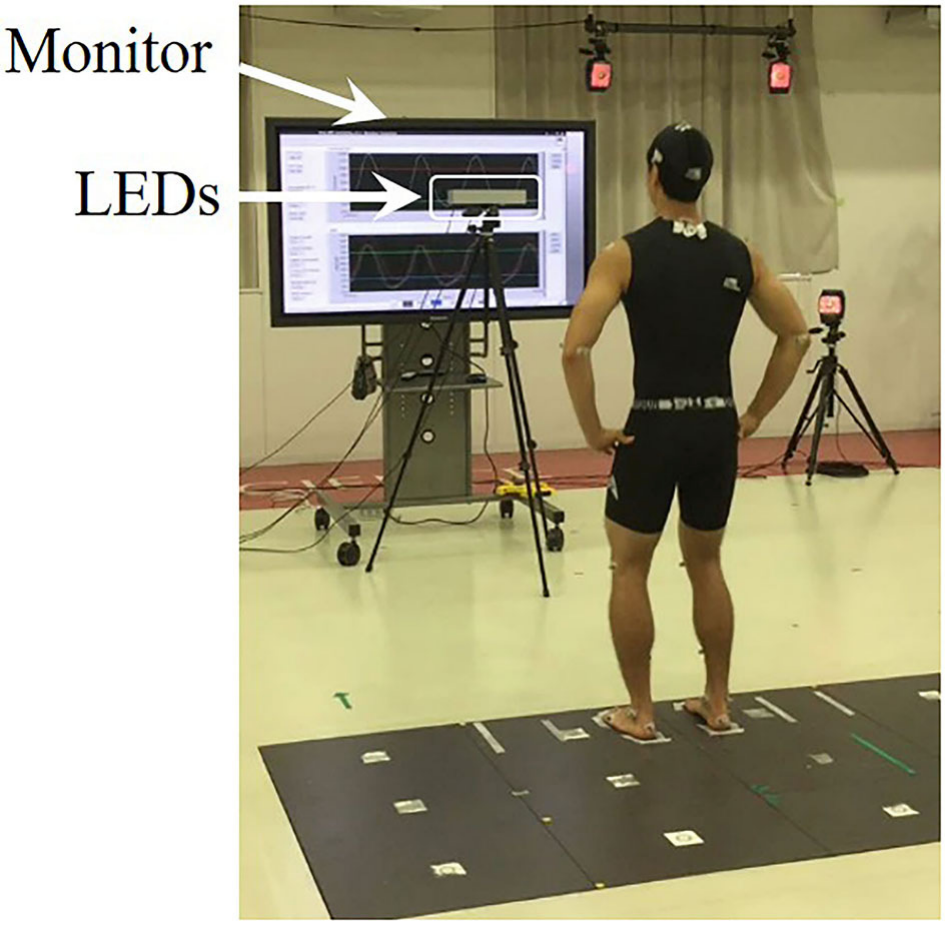
Experimental setup. The subject stood barefoot with an inter-malleolar distance of 10% of their body height (BH). Three sets of LEDs (left, center, right) at their eye level were used to provide a visual cue. They were asked to step laterally as rapidly as possible in response to the visual cue, at 10–20% BH from the lateral malleolus of the stepping foot. A monitor placed behind the LEDs provided real-time visual feedback of the vertical GRF in the P condition.

Figure 1 shows the experimental setup. A set of 39 reflective markers were placed on anatomical landmarks of each participant. In addition to the 28 marker placements previously reported (Hahn and Chou, 2004), 11 markers were placed on the 7th cervical vertebra (C7), posterior superior iliac spines (PSISs), medial humeral epicondyles, medial femoral epicondyles, medial malleoli, and 5th metatarsals. Each participant stood barefoot with their hands on their waist, with each foot on a separate force platform (Tec Gihan Company, Limited, Kyoto, Japan) and with an inter-malleolar distance of 10% of their body height (Sotirakis et al., 2016). The foot locations were traced onto the platform surface to ensure consistent initial foot placement over the trials. Three-dimensional marker trajectories were collected using a 16-camera motion capture system (Motion Analysis Corporation, Santa Rosa, CA) at 200 Hz. The trajectories were smoothed using a fourth-order low-pass Butterworth filter with a cut-off frequency of 8 Hz (Afschrift et al., 2019). Ground reaction forces (GRFs) were collected by the two force platforms at 1000 Hz and down-sampled to match kinematic data.

Three sets of light-emitting diodes (left, center, right LEDs) with an inter-LED distance of 10 cm were placed at 2 m in front of the participants at their eye level to provide a visual cue. The illumination of the center LED was used as a ready warning signal, followed by illumination of either the left or right LED go signal indicating the leg to step with in the lateral direction. A monitor was placed behind the LEDs to provide real-time visual feedback of the vertical GRF (vGRF). A real-time controller (NI cRIO-9033) with analog input and digital input/output modules (NI 9215 and NI 9402) was used for real-time signal processing through LabVIEW FPGA and LabVIEW Real-Time software (National Instruments, Austin, TX, USA).

### Procedures

The participants performed a rapid sidestepping task from a stationary position under the two-choice (left and right) reaction time conditions. The task was performed with preparatory movement [i.e., prep (P) condition] and without preparatory movement [i.e., no-prep (NP) condition]. They were instructed to step laterally as rapidly as possible in response to the visual cue at a distance of 10–20% of the body height from the lateral malleolus of the stepping foot as indicated by target lines on the platform surface. They took only one step with the leg indicated by the left or right LED go signal. Stepping distance was selected based on a previous investigation of lateral sidestepping, in which a stepping distance of 10–20 cm was used (Tateuchi et al., 2011). They were first familiarized with the task while looking at their foot placement before the experiment and were instructed to always look straight ahead at the monitor during the trials. In the NP condition, the participants were instructed to evenly distribute their weight between the limbs in the initial position to avoid a preparatory weight shift, which was confirmed by the vGRF monitored on the computer screen.

**Figure 2:**
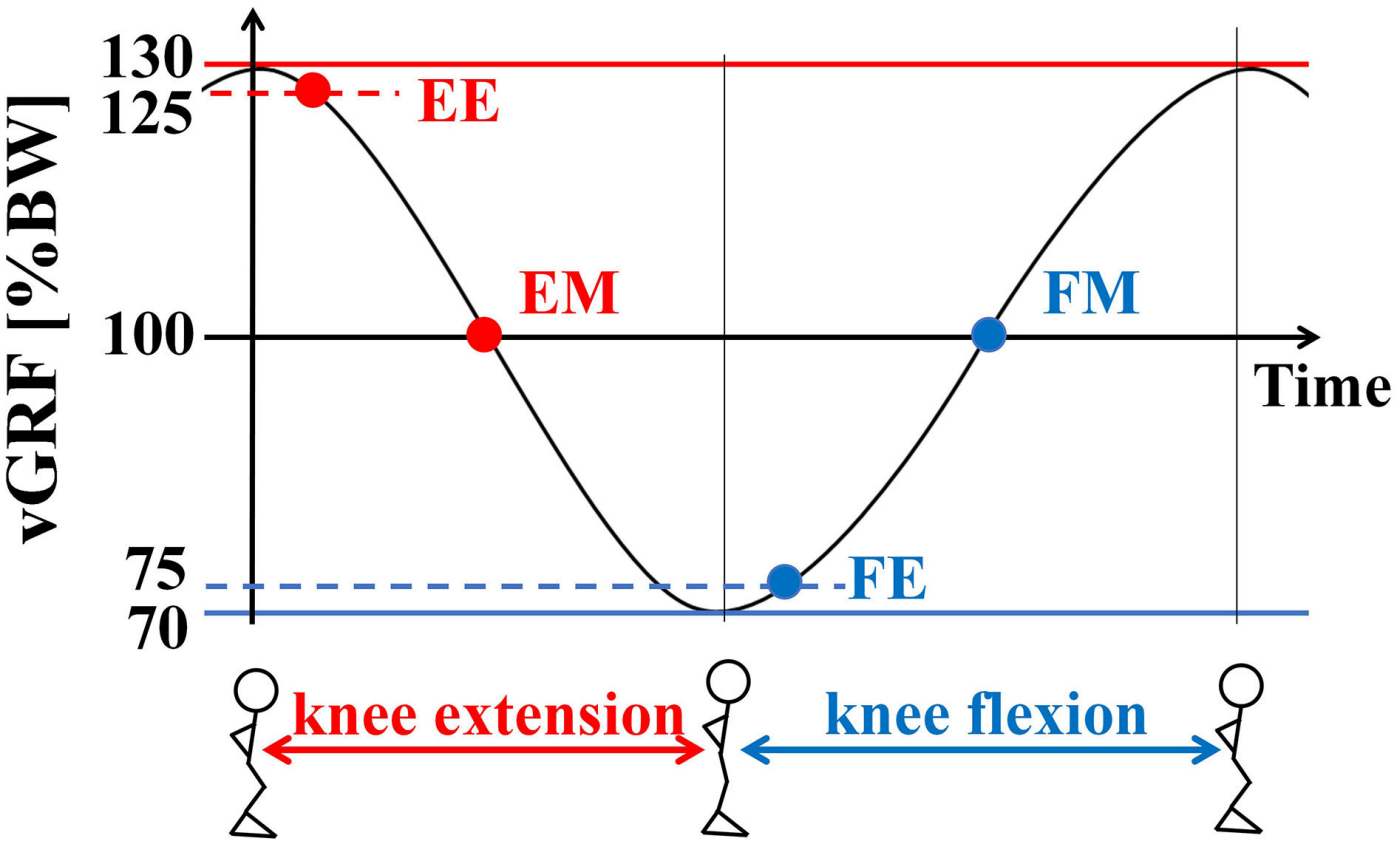
Visual cue presentation timing in the P condition. Real-time visual feedback of the vertical GRF (vGRF) was provided with the monitor behind the LEDs, and the subjects were asked to maintain the vGRF from 70 to 130% of their body weight (BW), followed by the sidestepping reaction task. The reaction signal was provided at the following four different loading instants while performing the preparatory movement: when the vGRF first fell below 125 and 100% BW during the knee extension phase (early phase: EE, and middle phase: EM), and when the vGRF first exceeded 75 and 100% BW during the knee flexion phase (early phase: FE, middle phase: FM).

In the P condition, the participants performed continuous alternating bilateral knee flexion-extension as preparatory movements. Real-time visual feedback of the vGRF was provided with the monitor behind the LEDs, and the participants were asked to maintain the vGRF from 70 to 130% of their body weight (BW) as indicated by target lines on the visual display, followed by the sidestepping reaction task. This corresponded with a knee flexion angle of 30–42° on average. The range of vGRF was selected to ensure that they were at unweighted (below 80% BW) and weighted (120% BW) states as defined in a previous investigation (Fujii et al., 2013). The reaction signal was provided at the following four different loading instants while performing the preparatory movement: when the vGRF first fell below 125 and 100% BW during the knee extension phase [early phase (EE) and middle phase (EM), respectively] and when the vGRF first exceeded 75 and 100% BW during the knee flexion phase [early phase (FE) and middle phase (FM), respectively] (Figure 2). These instants were selected to provide visual cues at the beginning of knee extension and flexion (EE and FE) and during knee extension and flexion (EM and FM), which corresponded with the largest and smallest loading instants and transition states between those instants, respectively. A metronome was used to control the timing of the preparatory movement cycle. The beat rate was set to 150 beats per minute, so that each movement cycle required 400 ms for completion (i.e., 200 ms for each knee flexion and extension phase) and the time interval between the visual cue instants was about 100 ms. This beat rate was determined through pilot experiments to be a comfortably achievable rate to ensure the range of vGRFs. Since the up-movement (knee extension on the beat) is more difficult to perform than the down-movement (knee flexion on the beat) (Miura et al., 2011), the subjects began the knee flexion phase on the metronome beat, i.e., down-movement.

For all trials, the center LED was first illuminated for 500 ms as a ready signal, followed by a go signal on either the left or right LED for 3 s after 1–4 s of randomized delay. The monitor was turned off before the ready signal for the P condition. Six trials were collected for each loading condition and direction. A total of 12 and 48 trials were collected in the NP and P conditions, respectively (NP: 2 directions × 6 trials; P: 2 directions × 4 loading conditions × 6 trials). The order in which the trials were presented was randomized, and the order of the P and NP conditions was counter-balanced between the subjects to minimize anticipation and sequence learning effects.

### Data Analysis

**Figure 3:**
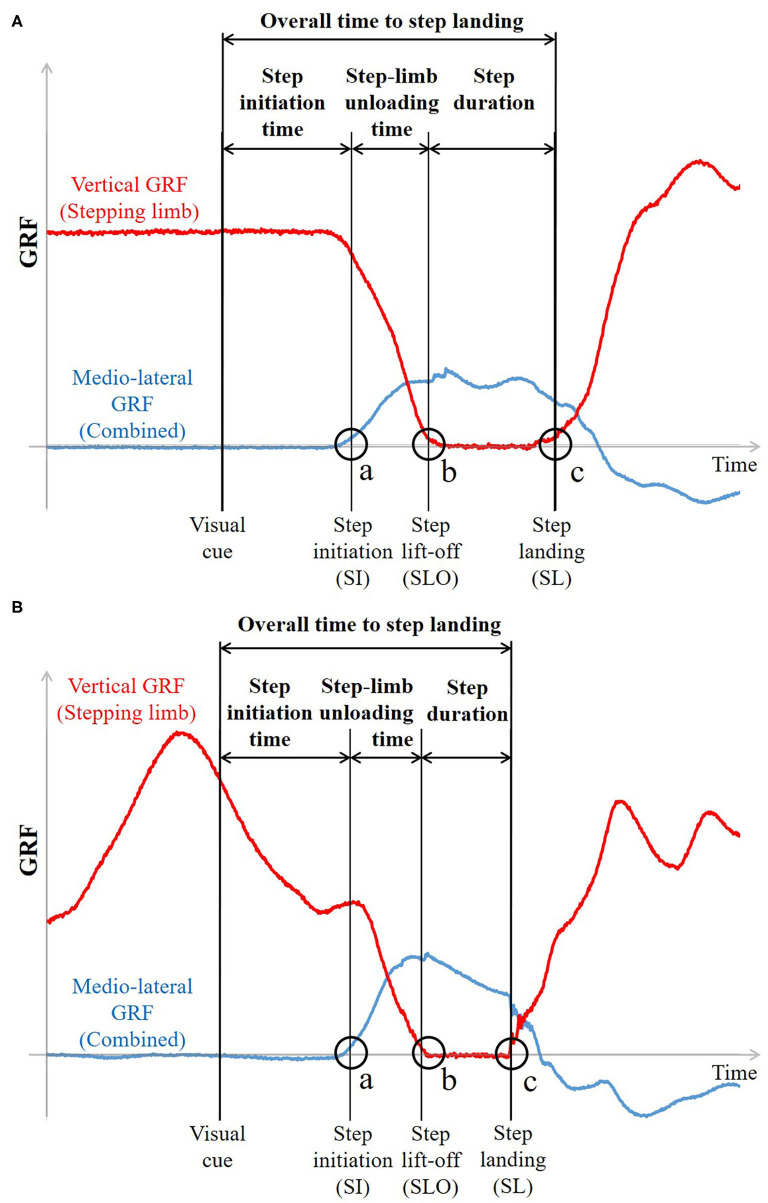
Representative time-history plots of mediolateral and vertical GRFs in **(A)** NP condition and **(B)** P condition (EE condition). (a) Step initiation (SI) was defined as the instant when the combined mediolateral GRF in the direction of the stepping limb side first exceeded 10N. (b,c) Step lift-off (SLO) and step landing (SL) instants were detected as the instants when the vertical GRF of the stepping limb first fell below 10N and exceeded 10N, respectively. SI time and SLO time were defined as the time interval from the visual cue to SI and to SLO, respectively. Step-limb unloading time and step duration were respectively defined from SI to SLO and from SLO to SL. Overall time to step landing was defined as the total response time from the visual cue until the instant of first step landing.

Step-initiation characteristics—step initiation time, step-limb unloading time, and step lift-off time—and step-execution characteristics—step duration, step velocity, step length, and overall time to step landing from the stimulus—were calculated for each side of stepping [non-dominant (ND) side and dominant (D) side] based on three-dimensional kinematic data and GRFs to assess sidestepping performance. The limb used to kick a ball was considered as the dominant limb (Paillard, 2017). Step initiation (SI) was defined as the instant when the combined mediolateral (ML) GRF in the direction of the stepping limb side last exceeded 10 N before step lift-off, indicating the onset of whole-body movement in the direction of stepping [Figure 3A(a) and Figure 3B(a) for NP and P conditions]. Since the onset of change in vertical and ML GRF has been reported to be simultaneous (Patla et al., 1993), SI was also treated as the onset of step-limb unloading. Step lift-off (SLO) and step landing (SL) instants were detected as the instants when the vGRF of the stepping limb first fell below 10 N with foot lift-off and then exceeded 10 N with foot landing, respectively [Figure 3A(b,c) and Figure 3B(b,c) for NP and P conditions]. Step initiation time and step lift-off time were defined as the time interval from the go signal to the SI and SLO, respectively. Step-limb unloading time and step duration were respectively defined from SI to SLO and from SLO to SL (Figure 3). Step velocity was calculated as the peak ML velocity of the step-limb ankle joint between SLO and SL. Step length was calculated as the ML displacement of the ankle joint of the stepping limb between SLO and SL. It was normalized to the stance width, calculated as the ML distance between the left and right ankle joints at the initial position. Overall time to step landing was defined as the total response time from the go cue until the instant of first step landing. Movement cycle duration was also calculated based on the vGRF as the time required to complete one preparatory movement cycle. It was averaged over the time interval between the ready signal and SI in the P condition. The loading condition at SI was calculated as the vGRF at SI expressed as % BW to assess the loading state at SI for each condition in the P condition. All data analyses were performed using MATLAB, version R2017b (MathWorks, Natick, MA, USA).

### Statistical Analyses

Considering step side (ND and D) and preparatory condition (NP, EE, EM, FE, and FM) as factors, we performed a two-way repeated-measures ANOVA with *post-hoc* tests with Bonferroni correction to examine the main and interaction effects on the sidestepping characteristics. When the sphericity assumption was violated, the Greenhouse-Geisser correction was applied. Indices of effect size, *r* (for pairwise comparisons), and partial eta-squared ($\eta_{p}^{2}$, for ANOVA) values were reported with *p*-values. Statistical analyses were performed using IBM SPSS Statistics for Windows, version 23 (IBM Corporation, Armonk, NY, USA). A significance level of *p* < 0.05 was used for all comparisons.

## Results

### Preparatory Movement Cycle Duration and Loading Condition at Step Initiation

The movement cycle duration for the preparatory movement was 395 ± 7 ms on average. The loading condition at step initiation for the P condition was 98 ± 13% of their BW for the EE condition, 113 ± 15% BW for the EM condition, 110 ± 16% BW for the FE condition, and 85 ± 8% BW for the FM condition on average.

### Step-Initiation Characteristics

Table 1 summarizes the results of two-way ANOVA for step-initiation characteristics. No significant interaction was observed between step side and preparatory condition in the step-initiation characteristics: step initiation time (*p* = 0.797, $\eta_{p}^{2}$ = 0.029), step-limb unloading time (*p* = 0.909, $\eta_{p}^{2}\;$ = 0.018), and step lift-off time (*p* = 0.854, $\eta_{p}^{2}$ = 0.023).

**Table 1:** Two-way ANOVA summary table for step-initiation characteristics.

**Variable**	**Source**	***df***	***F***	***p***	$\eta_{\text{p}}^{2}$	**Pairwise comparisons**
Step initiation time	Condition	1.7	10.0	< 0.001^*^	0.417	EE, EM < NP
	Side	1	13.1	0.003^*^	0.483	ND < D
	Condition × Side	4	0.4	0.797	0.029	
Step-limb unloading time	Condition	1.7	14.0	< 0.001^*^	0.500	FE, FM < NP, EE, EM
	Side	1	17.5	0.001^*^	0.555	D < ND
	Condition × Side	4	0.2	0.909	0.018	
Step lift-off time	Condition	1.7	14.9	< 0.001^*^	0.516	EE, EM, FE, FM < NP
	Side	1	1.1	0.314	0.072	
	Condition × Side	4	0.3	0.854	0.023	

* *p < 0.005*.

*EE and EM, early and middle phases of the knee extension phase; FE and FM, early and middle phases of the knee flexion phase; ND, non-dominant; D, dominant*.

**Figure 4:**
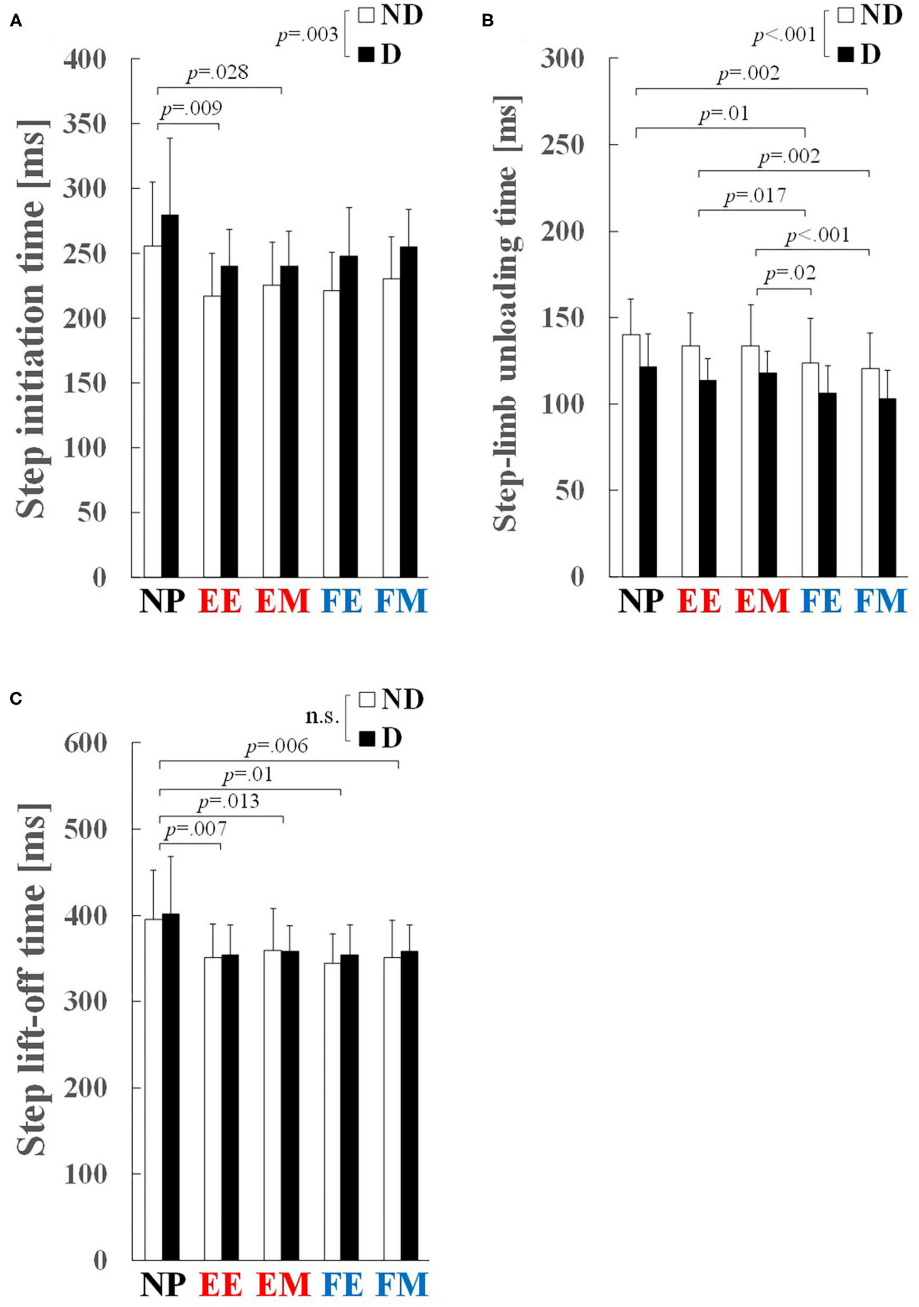
Step-initiation characteristics: **(A)** step initiation time, **(B)** step-limb unloading time, and **(C)** step lift-off time. Values and error bars are mean and SD. EE and EM, early and middle phases of the knee extension phase; FE and FM, early and middle phases of the knee flexion phase; ND, non-dominant; D, dominant.

A significant main effect of preparatory condition was observed for step initiation time (*p* < 0.001, $\eta_{p}^{2}$ = 0.417), step-limb unloading time (*p* < 0.001, $\eta_{p}^{2}$ = 0.500), and step lift-off time (*p* < 0.001, $\eta_{p}^{2}$ = 0.516). Pairwise comparisons revealed that step initiation time in the EE (*p* = 0.009, *r* = 0.75) and EM (*p* = 0.028, *r* = 0.70) conditions was significantly shorter by 13 and 15% than that in the NP condition, respectively (Figure 4A). Step-limb unloading time in the FE and FM conditions was significantly shorter than the NP (by 12 and 15%, *p* = 0.01, *r* = 0.74 and *p* = 0.002, *r* = 0.80), EE (by 7 and 10%, *p* = 0.017, *r* = 0.72 and *p* = 0.002, *r* = 0.80), and EM (by 9 and 11%, *p* = 0.02, *r* = 0.71 and *p* < 0.001, *r* = 0.85) conditions (Figure 4B). Step lift-off time in the EE (*p* = 0.007, *r* = 0.75), EM (*p* = 0.013, *r* = 0.73), FE (*p* = 0.01, *r* = 0.74), and FM (*p* = 0.006, *r* = 0.77) conditions was significantly shorter by 10–12% than in the NP condition (Figure 4C).

A significant main effect of step side was observed for step initiation time (*p* = 0.003, $\eta_{p}^{2}$ = 0.483) and step-limb unloading time (*p* = 0.001, $\eta_{p}^{2}$ = 0.555). Step initiation time on the ND side was shorter by 9% than that on the D side (230 ± 38 vs. 253 ± 40 ms; Figure 4A), and step-limb unloading time on the D side was shorter by 13% than that on the ND side (113 ± 17 vs. 130 ± 23 ms; Figure 4B). No significant main effect of step side was observed for step lift-off time (*p* = 0.314, $\eta_{p}^{2}$ = 0.072; Figure 4C).

### Step-Execution Characteristics

Table 2 summarizes the results of two-way ANOVA for step-execution characteristics. No significant interaction was observed between step side and preparatory condition in the step-execution characteristics: step duration (*p* = 0.824, $\eta_{p}^{2}$ = 0.026), step velocity (*p* = 0.625, $\eta_{p}^{2}$ = 0.045), step length (*p* = 0.763, $\eta_{p}^{2}$ = 0.024), and overall time to step landing (*p* = 0.936, $\eta_{p}^{2}$ = 0.014).

**Table 2:** Two-way ANOVA summary table for step-execution characteristics.

**Variable**	**Source**	***df***	***F***	***p***	$\eta_{\text{p}}^{2}$	**Pairwise comparisons**
Step duration	Condition	1.9	43.1	< 0.001^*^	0.755	EE, EM, FE, FM < NP
	Side	1	0.1	0.754	0.004	
	Condition × Side	2.3	0.4	0.824	0.026	
Step velocity	Condition	1.7	30.1	< 0.001^*^	0.682	EE, EM, FE, FM < NP
	Side	1	< 0.1	0.949	< 0.001	
	Condition × Side	4	0.7	0.625	0.045	
Step length	Condition	1.9	0.3	0.760	0.019	
	Side	1	< 0.1	0.313	0.072	
	Condition × Side	2.6	0.3	0.763	0.024	
Overall time to step landing	Condition	1.4	35.4	< 0.001^*^	0.716	EE, EM, FE, FM < NP
	Side	1	2.2	0.161	0.135	
	Condition × Side	4	0.2	0.936	0.014	

* *p < 0.005*.

*EE and EM, early and middle phases of the knee extension phase; FE and FM, early and middle phases of the knee flexion phase; ND, non-dominant; D, dominant*.

**Figure 5:**
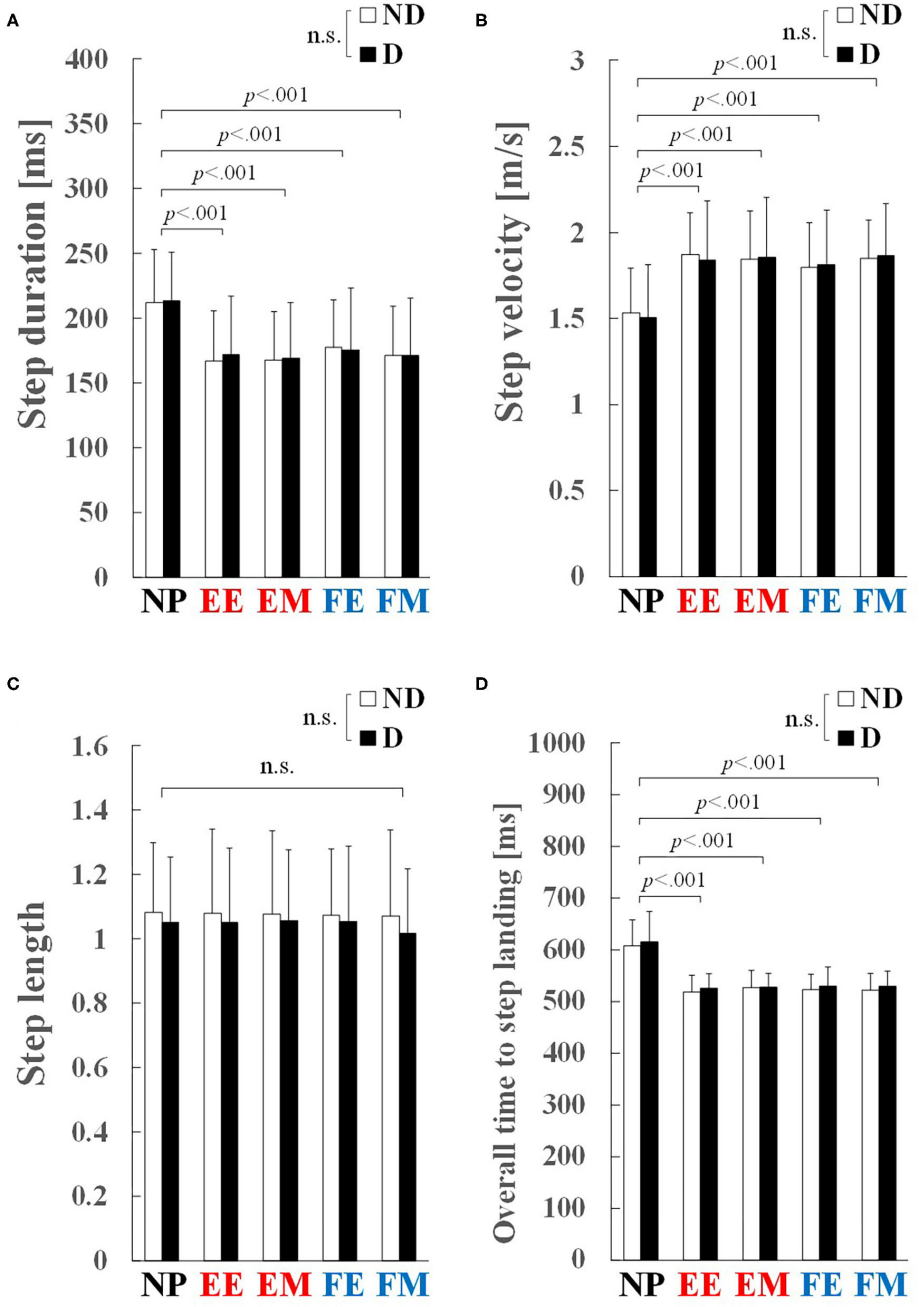
Step-execution characteristics: **(A)** step duration, **(B)** step velocity, **(C)** step length, and **(D)** overall time to step landing. Values and error bars are mean and SD. EE and EM, early and middle phases of the knee extension phase; FE and FM, early and middle phases of the knee flexion phase; ND, non-dominant; D, dominant. Step length was normalized to the stance width, and therefore is a unitless value.

A significant main effect of preparatory condition was observed for step duration (*p* < 0.001, $\eta_{p}^{2}$ = 0.755), step velocity (*p* < 0.001, $\eta_{p}^{2}$ = 0.682), and overall time to step landing (*p* < 0.001, $\eta_{p}^{2}$ = 0.716). Pairwise comparisons revealed that step duration in the EE, EM, FE, and FM conditions was significantly shorter by 17–21% than in the NP condition (*p* < 0.001, *r* ≥ 0.85; Figure 5A). Step velocity in the EE, EM, FE, and FM conditions was significantly larger by 19–22% than that in the NP condition (*p* < 0.001, *r* ≥ 0.84; Figure 5B). Overall time to step landing in the EE, EM, FE, and FM conditions was significantly shorter by 14–15% than that in the NP condition (*p* < 0.001, *r* ≥ 0.84; Figure 5D). No significant main effect of preparatory condition was observed for step length (*p* = 0.760, $\eta_{p}^{2}$ = 0.019; Figure 5C). No significant main effect of step side was observed in the step execution characteristics: step duration (*p* = 0.754, $\eta_{p}^{2}$ = 0.004; Figure 5A), step velocity (*p* = 0.949, $\eta_{p}^{2}$ < 0.001; Figure 5B), step length (*p* = 0.313, $\eta_{p}^{2}$ = 0.072; Figure 5C), and overall time to step landing (*p* = 0.161, $\eta_{p}^{2}$ = 0.135; Figure 5D).

## Discussion

Rection time sidestepping characteristics were compared among five different loading conditions: NP, EE, EM, FE, and FM. Step initiation time in the EE and EM conditions was shorter by 13 and 15%, and step-limb unloading time in the FE and FM conditions was shorter by 12 and 15% than in the NP condition, respectively. In contrast to our hypothesis, the preparatory movement shortened step lift-off time by 10–12% and step duration by 17–21% with 19–22% larger step velocity. This resulted in 14–15% shorter overall time to step landing from the go signal, regardless of the lower-limb loading conditions. These findings indicate that dynamic lower-limb loading and unloading with knee flexion-extension movements facilitated faster step lift-off, resulting from either faster step initiation timing or faster step-limb unloading time.

Step initiation time for the preparatory movement conditions was 235 ± 25 ms on average, and the overall movement cycle duration for the preparatory movement was 395 ± 7 ms. This result indicates that a step was initiated approximately half a cycle later after the visual cue presentation. More specifically, a step was initiated between FE and FM for the EE condition (98% BW), in between FM and EE for the EM condition (113% BW), in between EE and EM for the FE condition (110% BW), and in between EM and FE for the FM condition (85% BW). Thus, stepping was initiated during the knee flexion and extension phases that followed the visual cues presented during the knee extension (EE and EM) and flexion (FE and FM) phases, respectively. The phase-dependent improvements in the speed of step lift-off were differentially attributable to a faster step initiation interval than the no-prep condition when the go cue was presented during the preparatory knee extension (EE-EM) phase, compared with a faster step-limb unloading interval when the go cue was presented during the flexion (FE-FM) phase. It has previously been reported that an unweighted limb-loading state below 80% BW or a weighted state above 120% BW can shorten or delay sidestepping initiation (Fujii et al., 2013). Our findings further demonstrated that, rather than just/only the influence of discrete weighted or unweighted conditions, the continuously increasing and decreasing pre-step limb loading conditions accompanying knee flexion and extension affected sidestepping performance.

The facilitation of step initiation time was associated with increasing lower-limb loading during pre-step knee flexion preceding the transition to knee extension and step initiation. As the body weight must be transferred to the impending single stance limb for stepping to occur (Patla et al., 1993; Tateuchi et al., 2006; Sparto et al., 2014), pre-step loading of the stance limb would facilitate faster step initiation. The lower limbs were at the increasing loading state during the knee flexion phase (Figure 2), which appears to have facilitated faster support-limb loading and subsequent step initiation.

Compared with the facilitation of initiation timing with pre-step knee flexion, step-limb unloading time was shortened when the go cue was presented during the preparatory knee flexion (FE-FM) phase. This effect was associated with pre-step lower-limb unloading along with the knee extension movement at step initiation that resulted in shorter step-limb unloading time than the NP condition. Since the time to unload and lift the limb is shortened when the load is increased on the support leg (Patchay and Gahery, 2003; Aruin, 2006; Azuma et al., 2007; Shinya et al., 2009), preparatory bilateral knee extension movement appeared to assist unloading of the stepping limb by reducing the time to lift-off.

Forward or backward stepping are preceded by anticipatory postural adjustments (APAs) for lateral weight transfer involving step-limb loading and stance-limb unloading prior to step lift-off (Lepers and Breniere, 1995; Tateuchi et al., 2011; Sparto et al., 2014). Such interlimb loading-unloading of vertical GRFs indicative of APAs are small in amplitude and duration or even absent for lateral side stepping (Patla et al., 1993; Sparto et al., 2014). This indicates that stepping sideways can be quickly achieved by directly lifting the limb from the ground with step-limb knee flexion (step-limb take-off), while pushing off the ground with support-limb knee extension (support-limb push-off). Therefore, pre-step knee flexion preceding to support-limb knee extension to initiate a step facilitated faster support-limb push-off, while pre-step knee extension followed by step-limb knee flexion facilitated faster step-limb take-off, both resulting in faster step lift-off depending on the phase of the preparatory movement.

In addition to the effects of limb loading conditions on the initiation onset and step lift-off timing, we also found that the duration and velocity of first step execution phase between lift-off and ground contact were respectively shorter and greater with both flexion and extension preparatory movements compared with the NP condition. These results indicated that the first step initiation and execution timing were both dependent upon the limb loading conditions preceding and accompanying rapid stepping.

Although the rapid sidestepping performance was dependent on the conditions of limb loading at step onset, the preparatory movements that altered the contractile state of the limb musculature in advance of the step movement command may also have contributed to the results. For example, post-activation potentiation of the motor activity for stepping through alterations in motoneuronal excitability could have been enhanced by the prior flexion-extension movements (Hodgson et al., 2005). Additionally, because the preparatory knee movements involved alternating muscle lengthening and shortening contractions, the improvements in stepping could also have been associated with the stretch-shortening cycle phenomenon (Komi, 1984, 2000; Nicol et al., 2006). In this case, shortening of a muscle immediately after it is first lengthened potentiates muscular work and power, mainly due to stored elastic energy (Cavagna et al., 1965, 1968; Cavagna, 1977; Kubo et al., 1999; Komi, 2000; Nicol et al., 2006). Identifying the specific mechanisms underlying the observed pre-movement augmentation in sidestepping performance remains to be determined in future investigations.

Regardless of the lower-limb conditions, step initiation onset time was earlier for the non-dominant limb sidesteps, while the unloading time was shorter for dominant limb steps. This indicated that the loading and unloading phases were quicker on the dominant leg than non-dominant leg. Although such side-dependent differences were not found in the step lift-off time, the findings suggested that push-off and take-off with the dominant side could respectively reduce the time to initiate a step and unload the stepping limb.

Since both pre-step knee flexion and extension phases facilitate faster step lift-off, preparatory knee flexion-extension movements could be easily utilized in real game situations for more effective reactive stepping performance. For instance, the ability to accelerate rapidly from a stop or first step quickness to gain a step on a defender is important in initiating a drive for an attacker, while the defender needs to rapidly impede the attacker challenging the shot or pass in basketball games (Conrad, 2014). Bilateral knee flexion-extension movements as a preparatory action could be beneficial in such situations to facilitate effective movement initiation. Further studies are needed to determine if the preparatory movement would be applicable to real game situations to facilitate movement initiation.

Among the limitations of the study is the inclusion of only male collegiate basketball players. The findings may not be applicable to females and individuals who play other types of sports or to non-athletes. The experiment did not replicate real game situations and the findings were limited to sidestepping movement from a stationary position. Many real game situations require players to rapidly change directions while sprinting or in motion, as well as from a stationary start, i.e., a change-of-direction (COD) task (Hewit et al., 2013). Other means of preparatory movements with different initial steps taken, such as drop step, hip turn, backward step, and a pivot-crossover step, have been observed for the COD task (Dysterheft et al., 2013; Mccormick et al., 2014). Further investigation is needed to determine the effects of preparatory movement on COD performance with other types of stepping to be applicable to real game situations. The difference in initial posture may have also affected our results. The body center of mass (COM) was 3.5 cm (2% BH) higher in NP than P conditions at step initiation on average (57.3 ± 0.7% vs. 55.3 ± 0.8% BH). This could be the reason for slower step initiation in the NP condition. The effect of COM height on sidestepping performance needs to be determined in further studies. Finally, the preparatory movement cycle was paced with a specific rhythm in this study. Preparatory movements with different frequencies would likely affect stepping performance due to the interval between eccentric and concentric actions of a muscle for performance potentiation in the concentric phase of the SSC or other time-dependent physiological variables (Komi, 2000; Hodgson et al., 2005; Nicol et al., 2006). Further studies investigating the effect of different intervals and/or with other types of the preparatory movement such as side-to-side oscillations on stepping performance are warranted.

In conclusion, preparatory knee flexion-extension movements shortened subsequent step lift-off and step duration with a larger step velocity. Faster step lift-off was differentially attributable to either a faster step initiation onset or a shortened step-limb unloading interval from step initiation to step lift-off, depending on the phase of the preparatory movement. Pre-step lower-limb loading along with knee flexion facilitated faster step initiation, while pre-step lower-limb unloading along with knee extension facilitated faster step-limb unloading. Bilateral knee flexion-extension movements as a preparatory action could be utilized by invasion sports players to facilitate reactive stepping performance for more effective movement initiation.

## Data Availability Statement

The raw data supporting the conclusions of this article will be made available by the authors, without undue reservation.

## Ethics Statement

The studies involving human participants were reviewed and approved by The Ethics Committee of Ritsumeikan University Biwako-Kusatsu Campus, Japan. The patients/participants provided their written informed consent to participate in this study.

## Author Contributions

MF and EU contributed to conception and design of the work, data acquisition and analysis, interpretation of data, and manuscript preparation. AN, MR, and TI provided expertise and feedback on the work and reviewed and edited the manuscript. MF wrote the original draft. All authors contributed to the article and approved the submitted version.

## Conflict of Interest

The authors declare that the research was conducted in the absence of any commercial or financial relationships that could be construed as a potential conflict of interest.
